# Screening of Diabetic Retinopathy Using Teleophthalmology to Complement Human Resources for Eye Health: A Systematic Review and Meta-Analysis

**DOI:** 10.3390/clinpract12040050

**Published:** 2022-06-29

**Authors:** Thembile Zikhali, Chester Kalinda, Zamadonda Nokuthula Xulu-Kasaba

**Affiliations:** 1 Department of Optometry, School of Health Sciences, University of KwaZulu-Natal, Westville Campus, Durban 4000, South Africa; XuluKasabaZ@ukzn.ac.za; 2 Bill and Joyce Cummings Institute of Global Health, University of Global Health Equity (UGHE), Kigali 20093, Rwanda; KalindaC@ukzn.ac.za; 3 Institute of Global Health Equity Research (IGHER), University of Global Health Equity (UGHE), Kigali 20093, Rwanda; 4 Discipline of Public Health, College of Health Sciences, University of KwaZulu-Natal, Durban 4001, South Africa

**Keywords:** diabetic retinopathy, teleophthalmology, screening, blindness, diabetes

## Abstract

Diabetic retinopathy is a vascular disease of the retina that affects patients with uncontrolled diabetes. Untreated diabetic retinopathy (DR) can eventually lead to blindness. To date, diabetic retinopathy is the third leading cause of vision loss in the working class globally. Frequent retinal screening for all diabetic people is an effective method of preventing diabetic retinopathy blindness. This has relied on the use of ophthalmologists, but due to scarce resources, such as a shortage of human resources for eye health, this has denied many patients quality eye health care in a resource-limited setting. The recent advances on the use of teleophthalmology are promising to close this gap. This study aimed to map available evidence on the use of teleophthalmology in the screening of DR globally and to explore how this can be used to complement short-staffed eye clinics, especially in resource-constrained contexts. Studies were sourced from Google Scholar, PubMed, Science Direct, and EBSCO host. The final study selection was presented using a PRISMA chart. The mixed method appraisal tool was used to assess the quality of the nine studies included. The random effect model was used to estimate pooled prevalence estimates. Levels of heterogeneity were evaluated using Cochran’s Q statistic and I^2^. Of nine included studies, eight were from high-income countries. The screening was performed at the primary healthcare level in eight of nine included studies. Only one study used a mydriatic agent, and the commonly used fundus camera was the non-mydriatic fundus camera. The overall estimated pooled prevalence of DR was 29 (95%CI: 10–34). Teleophthalmology at the primary health care level showed that early intervention in diabetic retinopathy reduced avoidable blindness and ensured remote access to eye health professionals, thus alleviating the burden on them.

## 1. Introduction

The World Health Organization (WHO) describes diabetes mellitus (DM) as a metabolic disorder characterized and identified by the presence of hyperglycemia in the blood when left untreated [1]. The WHO and International Diabetes Federation (IDF) attributed the 0, 9% global increase from 420 million in 2014 to 424 million people with DM in 2017 [2] to the high burden of disease observed in adults living in low-to-middle-income countries (LMICs) [1]. This led to a projected 628 million people who would suffer from DM by 2045 if the condition is not adequately managed [2]. Of grave concern to eye health workers, however, are the associated sight-threatening complications that result from DM, further increasing the burden of avoidable blindness globally [3].

Diabetic retinopathy (DR) is a complication of DM that is defined as a chronic progressive, potentially sight-threatening disease of the retinal microvasculature associated with prolonged hyperglycemia [4]. DR affects a third of the population with DM, and, if left untreated, can lead to diabetic macular edema (DME), a common cause of blindness in diabetic people worldwide [2]. As the leading cause of new-onset blindness in industrialized countries and one of the leading causes of avoidable blindness globally, [2,5] the WHO recommended diabetic retinopathy screening (DRS) to be prioritized to ensure early detection, thus ensuring the early management and treatment of DR [6,7]. To further clarify DRS, the recommendation was to begin screening at the diagnosis of diabetes for type 2 DM, and five years post initial diagnosis and at puberty for the patient with type 1 DM [6,7]. Furthermore, DRS repetition was dependent on the stage of retinopathy that the patient was at in the initial screening. In the absence of retinal disease, DRS was recommended annually [2,3,8]. In patients with mild NPDR, DRS should be repeated every 6 to 12 months as a means of monitoring, whereas patients with severe NPDR, PDR, and DME must be referred to an ophthalmologist for treatment [8].

Early diagnoses and treatment of DM and DR are critical measures for preventing permanent blindness. Whilst DRS offers the solution of early detection, it is a challenge in under-resourced settings with poor access and connectivity for modern technology. Of further concern are the human resources for eye-health (HReH) needed to conduct DRS in under-staffed rural contexts, exacerbating the dire situation in most of sub-Saharan Africa (SSA) [9].

The International Council of Ophthalmology (ICO) and IDF identified retinal photography as the gold standard in DRS, largely preferred for its objective imaging and provision of a permanent record [2,10]. The Society for Endocrinology, Metabolism and Diabetes of South Africa (SEMDSA) further recommended that the ideal screening person should be an ophthalmologist or optometrist trained in detecting DR [7]. The diagnostic accuracy of teleophthalmology in the detection of DR was proven to be superior to many, owing to its 91% sensitivity in detecting referable DR and 92% specificity level [11]. This method has been successfully used in telemedicine, where various devices were proven to be effective in the detection of DR [12]. In the interest of early detection and effective management through DRS, this review aimed to answer the research questions: (1) What evidence is there on the use of teleophthalmology in the management of diabetic retinopathy? (2) How does the implementation of teleophthalmology complement the shortage of HReH? Emerging evidence will be key in that it will give direction on where most countries are with this method of DR screening and management. It will further assist in guiding policymakers and showing all economies, especially LMICs, how early screening and detection of DR can be incorporated into their health systems, and can assist in the alleviation of avoidable blindness through DRS.

## 2. Materials and Methods

This systematic review and meta-analysis were guided by the Arksey and O’Malley framework [13]. The framework focuses on five review stages, which are as follows: (a) identifying the research question, (b) identifying relevant studies, (c) study selection, (d) charting the data, and (e) collating and reporting results. The Preferred Reporting Items for Systematic Reviews and Meta-Analyses (PRISMA) chart was used to present study search results. The Population, Concept, and Context (PCC) framework, shown in Table 1, was used to determine the eligibility of the research question.

### 2.1. Study Selection and Eligibility Criteria

Literature searches were conducted using PubMed, Google Scholar, and EBSCO Host databases, namely, Health source: Nursing/Academic Edition, Health source—Consumer, CINAHL, and Academic Search Complete. Relevant Medical Subjects Headings (MeSH), as well as Boolean terms. “AND” was used to separate the areas of population, concepts, and context, while “OR” improved the search by separating synonyms. The search was conducted using specified keywords, such as: Diabetes mellitus OR diabetes AND ocular manifestations OR ocular complications OR retinopathy OR retinal diseases and telemedicine OR teleophthalmology. The literature search was carried out for the period of 1 January 2014 to 8 October 2019. This study period was chosen based on the first release of the International Council of Ophthalmology guidelines for Diabetic Eye Care, which was released in December 2013 [14] and would have begun to be implemented in January 2014. These guidelines summarize the screening, assessment, and treatment of diabetic eye disease worldwide. In South Africa, the General Ethical Guidelines for Good Practice in Telemedicine (GEGGPT) were also published in 2014 by the Health Professions Council of South Africa (HPCSA) [15].

The primary investigator (PI) conducted title screening to ensure that selected studies were of interest. This was followed by the abstract and full-text screening conducted by two independent reviewers. The eligibility assessment was guided by the following inclusion criteria: (1) studies published from 1 January 2014 to 9 October 2019; (2) studies on diabetic human subjects; (3) studies on both ocular manifestations of diabetes (DR) and teleophthalmology. Studies were excluded if they did not include evidence in both DR and teleophthalmology and if they were conducted on non-diabetic subjects. The protocol for this review exists and can be accessed through the following web address: http://www.ccsenet.org/journal/index.php/gjhs/issue/view/0/2333, accessed on 20 February 2022.

### 2.2. Quality Assessment

The Mixed Method Appraisal Tool (MMAT) version 2018 was used to determine the quality of studies included [16]. For the importance of quality in systematic reviews, the methodological rigor in this review was achieved by having two independent reviewers (ZXK and SHM) critically appraising the methodological validity of the included studies. This appraisal was guided by MMAT version 2018. As this tool does not recommend scoring, the scoring guideline of MMAT version 2011 was used [17]. Each of the five questions were rated 20% for each criterion met, making a total of 100% if all five criteria were met, and 0% if none of the criteria were fulfilled. For this review, the tool was used to evaluate (a) if the sampling strategy was relevant in addressing the research question; (b) whether the sample was representative of the target population; (c) whether the measurements were appropriate; (d) whether the risk of nonresponse bias was low; and (e) whether the statistical analysis was appropriate to answer the research question, as per section 4 of the tool.

### 2.3. Data Extraction

A standardized data charting tool was piloted and amended before being used. The tool extracted data on the following: author and date, study title, study aim, country of study, economic profiling, level of screening site, sample size, number of positive referrals, type of device used, a person taking the picture, professional to whom the picture was sent, mydriatic used, turnaround time, emerging themes, and significant findings.

### 2.4. Data Analysis

The random effect model was used to estimate the pooled prevalence estimates (PPE) and their 95% confidence intervals (CI) of diabetic retinopathy using MetaXL version 3.1 software. (http://www.epigear.com/ accessed on 12 March 2022). Considering the number of studies obtained, subgroup analysis was not conducted. The included studies were labeled utilizing tables and forest plots. The levels of heterogeneity were evaluated using Cochran’s Q statistic to evaluate the presence of heterogeneity. The I^2^ test was used to evaluate the amount of heterogeneity present. The I^2^ tests were categorized as low, moderate, and high if the I^2^ values were 25%, 50%, and 75%, respectively [18,19]. In addition, publication bias was assessed using the funnel and Doi plots. For the Doi plot, symmetry was determined using the Luis Furuya–Kanamori (LFK) index. The LFK index values within ±1, exceeding ±1 but within ±2, and exceeding ±2 were considered as having no asymmetry, minor asymmetry, and major asymmetry, respectively [20].

### 2.5. Ethical Consideration

No ethical approval was required as this review was carried out on published data that were already available in the public domain. The exemption from ethics for this review was granted by the UKZN Bio-medical Research Ethics Committee (BREC).

## 3. Results

### 3.1. Search Results

**Figure 1:**
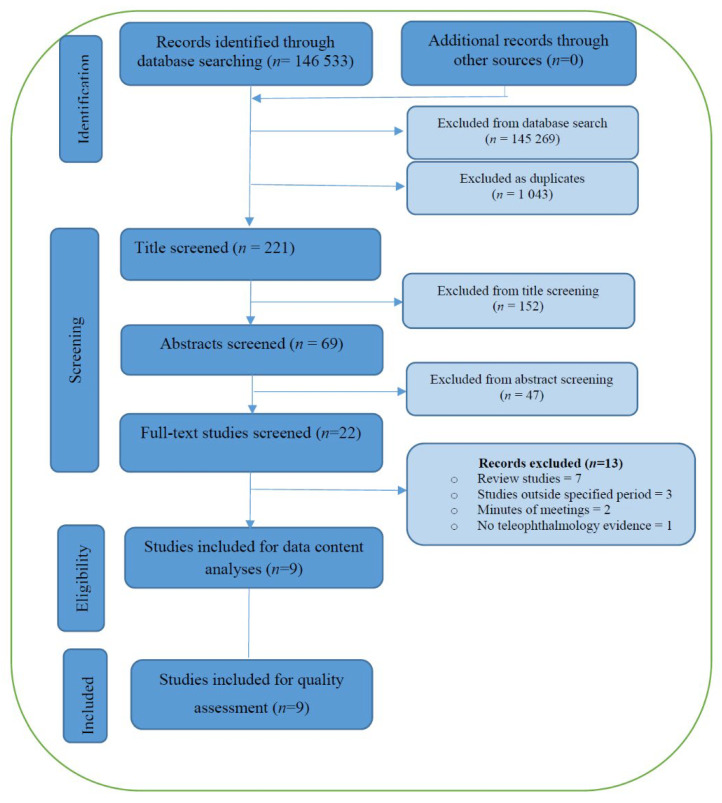
PRISMA flow diagram.

The electronic search strategy identified 146,533 articles, from which, 145,269 were excluded and 1043 duplicates were removed. The remaining 221 articles, stored in an endnote library created for this study, were title screened by the PI. This exercise culminated in the exclusion of 152 articles, leaving 69 studies to undergo abstract screening. Working independently, the PI and a trained co-screener then conducted abstract screening, ultimately agreeing on the removal of 47 as they did not meet the inclusion criteria (most studies were conducted before 2014). This resulted in 22 articles remaining for full text screening, which was also conducted by two independent co-screeners. After full-text screening, a further 13 articles were excluded for the following reasons: seven were review studies, two were minutes of meetings, three were conducted outside the specified period, and one did not present evidence on teleophthalmology. Therefore, nine articles met our inclusion criteria and were taken to the quality assessment and analysis stage. This process is displayed in Figure 1.

### 3.2. Characteristics of Included Studies

From the included studies, 322,725 participants were involved, where 97,042 were true positives, identified at the DR screening level. The nine studies included had the following listed aims:

- Evaluating the ability of trained imagers in using the ultra-wide field fundus camera (UWF) to determine the presence or absence of DR.
- Evaluating the relative diagnostic value of the non-mydriatic fundus photographer (NMFP).
- Evaluating telemedicine screenings for DR among patients with type 1 or 2 diabetes in rural areas.
- Describing the telemedicine reporting of DR screening using UWF.
- Describing the results of DR screening programs implemented in primary care areas.
- Characterizing the prevalence of DR and DME.
- Exploring the feasibility of using telemedicine for DR screening.
- Identifying the prevalence and risk factors of DR and comparing DR identification and ungradable image rates between non-mydriatic NMFP and UWF.

### 3.3. Features of DR Screening

All included studies did not use a mydriatic agent, except one study, which used a single drop of 1% tropicamide before screening [21]. Retinal images were taken by certified imagers in three studies, [22,23,24], trained nurses in two studies [25,26], physicians in one study [27], and a combination of nurses and physicians in another study [21], and ultimately by a combination of nurses and clinic technicians in the last study [28]. One study did not specify a retinal imager [29]. The retinal images were graded by optometrists in three studies [22,23,24], graded by an ophthalmologist in five [21,26,27,28,29], and by primary care physicians in one study [25]. Screening devices were generally fixed, and only one study used a Topcon portable fundus camera [25]. The camera type used in most studies was NMFP (seven studies), UWF was used in one study, and a combination of NMFP and UWF in another study. The commonly used camera model was Topcon TRC–NW6S.

Of the included studies, eight were from high-income countries, with five conducted in the United States of America (USA). Only one included study was from LMICs. From most of the included studies, DRS was conducted at the primary health care level, whereas only one study was conducted at a private multi-specialty hospital. The characteristics of the included studies are shown in Table 2.

**Figure 2:**
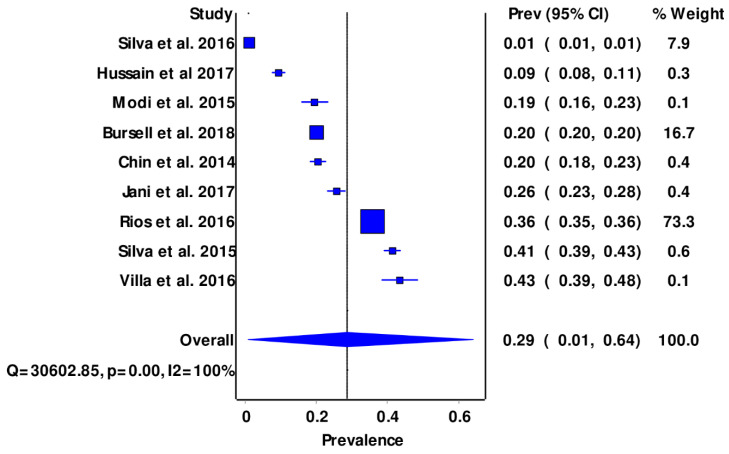
Forest plots showing pooled prevalence of DR.

The overall estimated pooled prevalence of diabetic retinopathy among the selected studies was 29 (95% CI: 10–64%) (Figure 2), and a high level of heterogeneity was observed in the selected studies (Supplementary File).

## 4. Discussion

This study aimed to identify available evidence on the use of teleophthalmology for the early diagnoses and management of DR. Further, it sought to identify health workers that were used in imaging, grading, and treatment, as the shortage of health workers was known to be a global problem.

A total of nine studies on diabetic retinopathy and teleophthalmology met the inclusion criteria. The overall estimated pooled prevalence of diabetic retinopathy among the selected studies was 29 (95% CI: 10–64%). This was lower than the previously reported global prevalence values of 34.6% in 2012 [30,31]. Similarly, the study finding was consistent with the decline in DR prevalence reported in the USA and other industrialized countries for patients with type 1 DM [6,32]. The decline in DR prevalence in HICs was believed to be the result of the enhanced management protocols of DM in resource-rich countries, where systems were initiated to ensure the implementation of early DRS and information availability [9,33]. Furthermore, the low prevalence rate from this study could be attributed to the reality that most of the included studies were conducted in high-income countries, where there were more HReH at the primary health care level, contrary to the case in LMICs [34,35,36]. It follows, then, that the prevalence of DR from the current study was found to be lower than that in SSA countries such as Tanzania (27.9%) and Zimbabwe (28.4%) [35,36]. Anecdotal evidence from WHO reported that the prevalence of DM and DR was on the increase in LMICs, whereas it showed a decline in HICs [6].

Considering that most of the included studies were from resource-rich economies, it follows that the use of DRS has been widely implemented in resource-rich countries, whereas limited-resource countries have fallen behind. It is further evident that increased DRS is positively associated with reduced DR. To mitigate the risk of DM in LMICs, programs implementing the improvement of screening need to be prioritized, more so at primary health care levels, to ensure an improvement in DRS uptake in these communities [37,38,39].

The current systematic review highlighted the effectiveness of non-ophthalmic health workers in constructive task shifting following relevant training. These health workers were trained in fundus photography and went on to produce gradable pictures. Similar findings were reported in previous studies, where the non-ophthalmic workforce proved competency, following training, and produced gradable pictures that were shared as tele-retinal images for DR grading [21,25,27]. Hussain and Jani et al. further explained that trained nurses were effective at taking accurate retinal images sufficient for grading [26,28]. Other authors affirmed this, explaining that training is key in the implementation of teleophthalmology at the PHC level, where medical personnel in those facilities were also effective in retinal imaging [24,29,40]. This task shifting to nurses and other cadres merely reduced the workload from optometrists and ophthalmologists, sparing them for interpreting images and grading the level of DR, as well as the management thereof [21,41]. In contrast, Bouskill et al. said that this method only caused a swing in traditional roles and reduced the workload from ophthalmic cadres, whilst adding that very load to other cadres who were already overworked in their areas of skill [41].

The actual interpreting or grading was conducted by optometrists and ophthalmologists in most studies, further reducing the workload of ophthalmologists. This review showed that trained primary health care providers such as optometrists can facilitate teleophthalmological DRS and grading, whilst ensuring accurate and expedited triage and leaving ophthalmologists for management. This strategy, in most cases, could reduce turnaround times for ophthalmologists. An Australian study reported that optometrists were effective at diagnosing and facilitating the management of DR, in collaboration with ophthalmologists for further management [42]. Recent advances have advocated the use of teleophthalmology for DRS, using fundus cameras, whose accuracy has been reported to be most ideal for grading clinicians [22,23].

This study further continued to analyse camera types and their properties following data extraction from the included studies. Two camera types were used in the studies: a non-mydriatic fundus photograph (NMFP) and the ultra-widefield (UWF) camera. Both units worked well without mydriasis, ensuring prompt and efficient patient service. In most of the included studies, the NMFP camera was used. The most gradable images, owing to a higher specificity and sensitivity, appeared to have been those captured using a UWF camera, which Silva et al. went on to describe as a fundus camera of superior imaging quality when compared with NMFP [23]. The benefits of the UWF were that it required minimal training compared to NMFP and produced optimal images through media opacities and small pupils, whilst being easily adaptable for use by people from all population backgrounds [22]. On the contrary, its cost was markedly higher than that of the NMFP, a disadvantage for health systems that are usually budget-constrained [23]. Of note, however, was the cumbersome nature of both machines as they were fixed and not portable, offering limited movability for inter-facility use in poorly resourced settings. The consensus was unanimous from most studies, however, in that the UWF could be used for DR screening in both urban and rural settings, owing to its ease of adaptability to different colours and condition of eyes, and its ease of training to all health workers [27]. The likely reason for which most studies probably used the NMFP in this instance is most likely its affordability concerning the UWF. This not only saved costs for the health system, but also saved time for patients by cutting out the time they could have waited for pupillary dilation before fundus examination [43].

Albeit easier to use, producing ideal images with its diverse adaptation, the UWF camera would be a beneficial investment if it were affordable. Large patient numbers would be screened at a rapid rate, with a high level of accuracy. In reality, however, the NMFP is advantageous as it is a cheaper unit that still takes gradable fundus images. In that light, it is recommended, as it would enable funders to equip more health facilities on a minimal budget. Further, the intervention would aid in early treatment, and ultimately, a reduction in DR disease development.

The main limitation of this study was that most of the reviewed articles represented one income group/economical profile. It would have been beneficial for policy formulation to explore how poor economies used teleophthalmology for DR. Moreover, none of the included articles discussed details on image transfer and storage, information that could have been used in implementing teleophthalmology, especially in LMICs. Finally, only one study detailed a standardized screening method; this would have been useful, as this would have helped to develop improved protocols.

## 5. Conclusions

This systematic review affirmed that DR prevalence had decreased since 2018 and is now at a prevalence of 29%. Teleophthalmology has been used in the management of DR, more in well-resourced countries than LMICs. The grading of fundus images was conducted mainly by ophthalmologists (some specialized) and trained optometrists, ensuring task sharing and the improved co-management of cases between the two cadres. Other tasks, such as taking images, were largely conducted by the easily accessible non-ophthalmic health workforce, a strategy that could be used to bridge the gap in HReH shortage. The UWF had a superior image quality, yet was costly. Due to the cost, the NMFP was the preferred device. The implementation of DRS could be valuable if implemented in LMICs such as SSA, as these have shown a dire shortage of HReH. Furthermore, the implementation of teleophthalmology for DR screening at primary levels of care could potentially improve the quality of eye care and prevent avoidable blindness. Evidence has shown that DRS ensured significant improvements in early detection, diagnosis, and early treatment outcomes for patients with severe retinal damage. The use of teleophthalmology is recommended, as it would enhance health care systems and reduce the global burden of diabetic blindness, as ophthalmologists would access their cases remotely and be able to provide timely care.

## Figures and Tables

**Table 1:** PCC model.

Population	Diabetic Patients, Ocular Manifestations of Diabetes, Ocular Complications of Diabetes, Diabetic Retinopathy, Retinal Complications of Diabetes
Concept	Tele-ophthalmology or Telemedicine
Context	All available studies (globally)

**Table 2:** Characteristics of nine included studies matching eligibility criteria, presenting evidence on diabetic retinopathy and the use of teleophthalmology.

Author, Year	Country, Economical Profiling	Study Design	Level of Care (PHC/Other)	Type of Health Provider Taking Image	Professionals Grading Images	Device Used, Fixed/Portable	Mydriatic Used [Yes/No]	Sensitivity Level
Chin, 2014 (21)	United States of America (USA) HIC	Retrospective, Cross-sectional (C/S) study	Primary Health Care (PHC)	Non-Ophthalmologist (NON) [Trained medical personnel]	Ophthalmologist (Retinal Specialist)	Non mydriatic fundus photography (NMFP), fixed	No	20.40%
Modi, 2015 (22)	India LMIC	Quantitative, C/S study	PHC	Not specified	Ophthalmologist (Retinal specialist)	NMFP, fixed	No	19.40%
Silva, 2015 (23)	USA HIC	Quantitative, C/S study	PHC	NON (Trained imagers)	NON (Optometrist)	Ultra-wide Field photographer (UWF), fixed	No	41.30%
Silva, 2016 (24)	USA HIC	Retrospective, C/S study	PHC	NON (Trained imagers)	NON (Optometrist)	NMFP and UWF, fixed	No	0.90%
Rios, 2016 (25)	Spain HIC	Retrospective, C/S study	PHC	NON (Nurses and Physicians)	Ophthalmologist	NMFP	Yes	35.60%
Villa, 2016 (26)	Spain HIC	C/S study	PHC	NON Trained Nurses	NON (PHC Physicians)	NMFP, portable	No	43.45%
Jani, 2017 (27)	USA HIC	C/S study	PHC	NON Trained Nurses and Clinic Technician	Ophthalmologist	NMFP, fixed	No	25.50%
Bursell, 2017 (28)	USA HIC	C/S study	PHC	NON (Trained imagers)	NON (Optometrist)	NMFP, fixed	No	20.00%
Hussain, 2017 (29)	United Arab Emirates HIC	C/S study	Multi-Specialty	NON (Trained Nurses)	Ophthalmologist (Retinal specialist)	UWF, fixed	No	9.20%

## Data Availability

The data analyzed in this manuscript are readily available in public domain.

## Supplementary Materials

The following supporting information can be downloaded at: https://www.mdpi.com/article/10.3390/clinpract12040050/s1, Figure S1A: Doi plot showing; Figure S1B: Funnel plot showing: publication bias.
