# Supplementary material for: Screening of Diabetic Retinopathy Using Teleophthalmology to Complement Human Resources for Eye Health: A Systematic Review and Meta-Analysis
Source: Clin Pract. 2022 Jun 29;12(4):457–67. doi: 10.3390/clinpract12040050 (PMC9326517; doi:10.3390/clinpract12040050)
Supplement: Supplementary file 1 [file clinpract-12-00050-s001.zip › clinpract-1737908-supplementary.pdf]

Supplementary file 1A

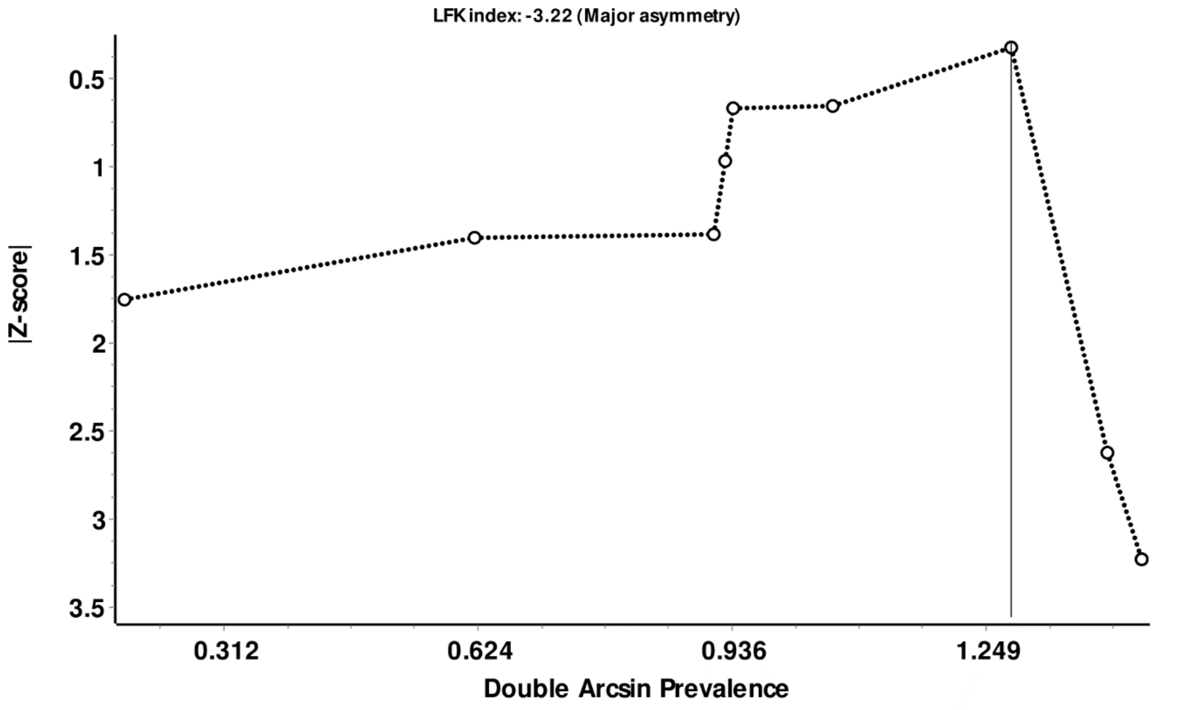

Figure S1A: Doi plot showing:

Supplementary file 1B

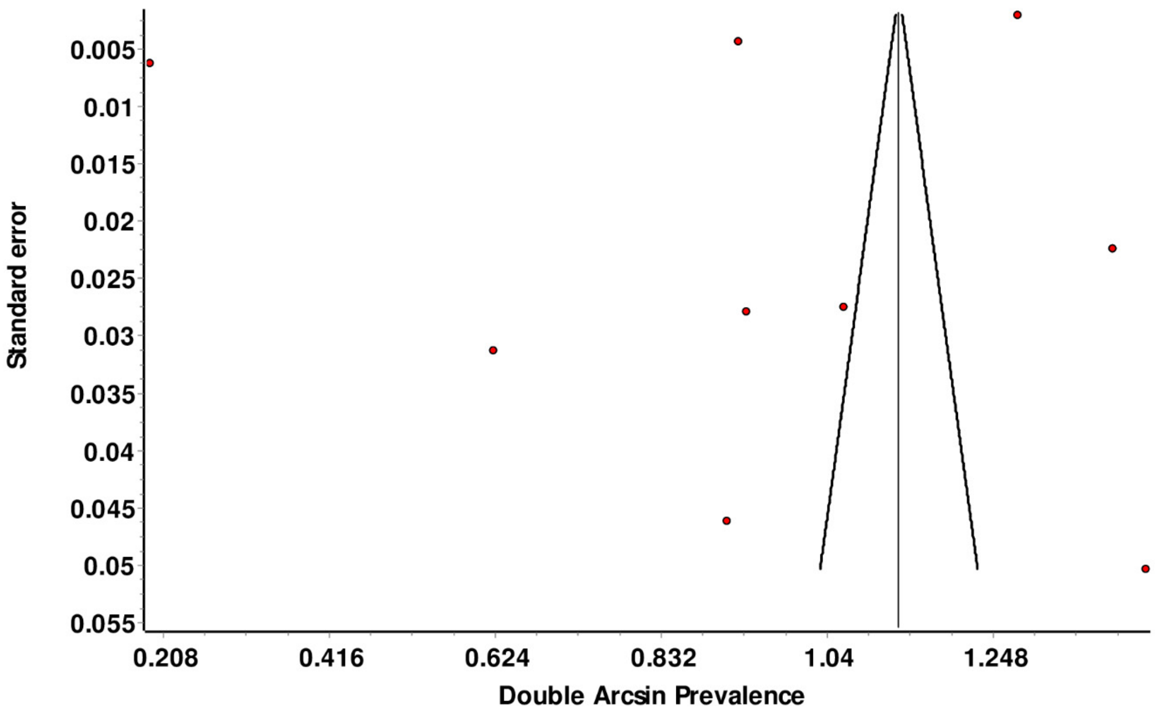

Figure S1B: Funnel plot showing: publication bias
